# Supplementary material for: Mapping the evolution of fertility support policies in China: A content and instrumental analysis
Source: PLoS One. 2025 Oct 9;20(10):e0332137. doi: 10.1371/journal.pone.0332137 (PMC12510515; doi:10.1371/journal.pone.0332137)
Supplement: S1 Appendix — (ZIP) [file pone.0332137.s001.zip › S1 Appendix. 226 original policy documents/125-外商投资企业劳动管理规定(FBM-CLI-4-10571).doc]

外商投资企业劳动管理规定

**发布部门：** [劳动和社会保障部(含劳动部)(已撤销)](https://www.pkulaw.com/law/chl?Aggs.IssueDepartment=60212) [对外贸易与经济合作部(含原对外经济贸易部)(已变更)](https://www.pkulaw.com/law/chl?Aggs.IssueDepartment=60232) [机构沿革](javascript:void(0))

**发文字号：**劳部发〔1994〕246号

**发布日期：**1994.08.11

**实施日期：**1994.08.11

**时效性：** [失效](https://www.pkulaw.com/law/chl?Aggs.TimelinessDic=02)

**效力级别：** [部门规章](https://www.pkulaw.com/law/chl?Aggs.EffectivenessDic=XE0301)

**法规类别：** [外商投资企业劳动人事工会](https://www.pkulaw.com/law/chl?Aggs.Category=04707) [劳动工会综合规定](https://www.pkulaw.com/law/chl?Aggs.Category=08701) [营商环境优化](https://dobusiness.pkulaw.com/)

**失效依据：**[劳动和社会保障部关于废止部分劳动和社会保障规章的决定](javascript:TMP(99542,0,'chl'))

劳动部、对外贸易经济合作部关于印发
《外商投资企业劳动管理规定》的通知
（劳部发〔１９９４〕２４６号）

各省、自治区、直辖市、经济特区及计划单列市劳动（劳动人事）厅（局）、对外经济贸易委员会（厅、局），海南省经济合作厅、湖南省招商局、上海市、厦门市外资委、深圳市外资办、哈尔滨市外资局、国务院各部门、总公司、计划单列企业集团劳动工资司（处）：
　　现将《外商投资企业劳动管理规定》印发给你们，请结合本地情况贯彻执行。
　　　　　　　　　　　　　　　　　　　　　　　　一九九四年八月十一日
　　　　　　　　　　　　外商投资企业劳动管理规定

　　**第一条**　为了保障外商投资企业（以下简称企业）及其职工的合法权益，确立、维护和发展企业与职工之间稳定和谐的劳动关系，根据国家法律、行政法规，制定本规定。

　　**第二条**　本规定适用于中华人民共和国境内设立的中外合资经营企业、中外合作经营企业、外资企业、中外股份有限公司及其职工。

　　**第三条**　县及县以上各级人民政府的劳动行政部门依据本规定，对企业的用人、培训、工资、保险福利待遇和劳动安全卫生等实行监察。

　　**第四条**　企业制定的规章制度，不得违反国家的法律、行政法规。

　　**第五条**　企业按照国家有关法律、行政法规，自主决定招聘职工的时间、条件、方式、数量。

　　企业招聘职工，可在企业所在地的劳动部门确认的职业介绍中心（所）招聘。经当地劳动行政部门同意，也可以直接或跨地区招聘。

　　企业不得招聘未解除劳动关系的职工。禁止使用童工。

　　**第六条**　企业招聘职工时，应当在中国境内招聘中方职工；确需招聘外籍及台湾、香港、澳门地区人员的，必须按照国家有关规定，经当地劳动行政部门批准，并办理就业证等有关手续。

　　**第七条**　企业应当建立职业培训制度，对职工进行职业培训。对从事技术工种或有特殊技能要求的职工，须经过培训后，持证上岗。培训经费须按照国家有关规定提取和使用。

　　**第八条**　劳动合同由职工个人同企业以书面形式订立。工会组织（没有工会组织的应选举工人代表）可以代表职工与企业就劳动报酬、工时休假、劳动安全卫生、保险福利等事项，通过协商谈判，订立集体合同。

　　劳动合同、集体合同的内容，应符合国家有关法律、行政法规。

　　**第九条**　劳动合同签订后，应当于一个月内到当地劳动行政部门鉴证。集体合同订立后，应报送当地劳动行政部门备案。劳动行政部门自收到之日起１５日内未提出异议的，集体合同即行生效。

　　**第十条**　劳动合同期满或双方约定的终止条件出现，劳动合同即行终止。经双方同意，可以续订劳动合同。

　　劳动合同变更需经双方协商同意，并办理劳动合同变更手续。劳动合同变更内容，可由劳动合同双方商定。

　　**第十一条**　有下列情形之一的，企业或职工可以解除劳动合同：

　　（一）劳动合同当事人协商一致；

　　（二）试用期内不符合录用条件、职工不履行劳动合同、严重违反劳动纪律和企业依法制定的规章制度，以及被劳动教养或被判刑的，企业可以解除劳动合同；

　　（三）企业以暴力、威胁、监禁或者其他妨害人身自由的手段强迫劳动；企业不履行劳动合同或者违反国家法律、行政法规，侵害职工合法权益的，职工可以解除劳动合同。

　　**第十二条**　有下列情形之一的，企业在征求工会意见后，可以解除劳动合同，但应提前３０日以书面形式通知职工本人：

　　（一）职工患病或非因工负伤，医疗期满后，不能从事原工作或不能从事由企业另行安排的工作的；

　　（二）职工经过培训、调整工作岗位，仍不能胜任工作的；

　　（三）劳动合同订立时所依据的客观情况发生变化，致使原劳动合同无法履行，经双方协商不能就变更劳动合同达成协议的；

　　（四）法律、行政法规规定的其他情形。

　　**第十三条**　职工患职业病或因工负伤并被确认丧失或部分丧失劳动能力的，职工患病在规定的医疗期内的，女职工在孕期、产期、哺乳期内的，用人单位不得解除劳动合同。因患职业病或因工致残的职工，若本人要求解除劳动合同，企业应按当地政府规定，向社会保险机构缴纳因工致残就业安置费。

　　职工患病或非因工负伤的医疗期限按现行规定执行。

　　**第十四条**　企业的工资分配，应实行同工同酬的原则。职工工资水平应在企业经济发展的基础上逐年提高。企业职工的工资水平由企业根据当地人民政府或劳动行政部门发布的工资指导线，通过集体谈判确定。

　　职工法定工作时间内的最低工资，不得低于当地最低工资标准。

　　**第十五条**　企业必须以货币形式按时足额支付职工工资，每月至少要支付一次，并为职工代扣、代缴个人所得税。

　　**第十六条**　企业应当按照有关规定进行劳动工资统计，并向所在地区劳动行政部门、财政部门及统计部门和企业主管部门报送劳动工资统计报表。

　　**第十七条**　企业必须按照国家有关规定参加养老、失业、医疗、工伤、生育等社会保险，按照地方人民政府规定的标准，向社会保险机构按时、足额缴纳社会保险费。保险费应按照国家规定列支。职工个人也应按照有关规定缴纳养老保险费。

　　**第十八条**　企业应当建立职工《劳动手册》和《养老保险手册》制度，记录职工的工龄、工资及养老、失业、工伤、医疗等社会保险费用的缴纳与支付情况。

　　**第十九条**　企业对依照本规定第十一条第一、三款、第十二条规定解除劳动合同的职工，应当一次性发给生活补助费。对依照本规定第十二条一款规定解除劳动合同的，除发给生活补助费外，还应当发给医疗补助费。

　　**第二十条**　生活补助费和医疗补助费标准，根据其在本企业的工作年限计算。生活补助费按照每满１年发给相当本人１个月的实得工资；医疗补助费按在本企业工作不满５年的，发给相当本人３个月的实得工资，５年以上的为６个月实得工资。在本企业工作６个月以上不满１年的，按１年计算。

　　生活补助费和医疗补助费计发基数，按本人解除劳动合同前半年月平均实得工资计算。

　　**第二十一条**　企业按照有关规定宣布解散或经双方协商同意解除劳动合同时，对因工负伤、或者患职业病经医院证明正在治疗或疗养、以及医疗终结经劳动鉴定委员会确认为完全或者部分丧失劳动能力的职工，享受抚恤待遇的因工死亡职工遗属，在孕期、产期和哺乳期的女职工，以及未参加各项社会保险的职工，应当根据企业所在地区人民政府的有关规定，一次向社会保险机构支付所需要的生活及社会保险费用。

　　**第二十二条**　企业职工在职期间的福利待遇，按照国家有关规定执行。

　　**第二十三条**　企业应当按照当地人民政府的规定，提取使用中方职工住房基金。

　　**第二十四条**　企业职工享受国家规定的节假日、公休假日、探亲假、婚丧假、女职工产假等假期。

　　**第二十五条**　企业因订立集体合同与工会或工人代表发生争议，争议双方协商不能解决的，可以由当地劳动行政部门组织争议双方协商处理；企业因履行集体合同发生的争议，经双方协商不能解决的，可以依法申请仲裁、提起诉讼。

　　**第二十六条**　企业的劳动争议、劳动安全卫生、工伤事故报告和处理、工作时间、女职工和未成年工的特殊保护等，按国家规定执行。

　　**第二十七条**　企业或者职工一方违反劳动合同，侵害对方利益，给对方造成损失的，应当承担赔偿责任。

　　**第二十八条**　企业违反本规定招聘职工的，当地劳动行政部门对企业可以按被招聘者月平均工资的５－１０倍处以罚款，并责令其退回招聘的职工。

　　**第二十九条**　企业职工工资低于当地最低工资标准的，由当地劳动行政部门责令其限期纠正，企业除按最低工资标准补齐外，还应按实发工资与最低工资标准差额的２０％－１００％发给职工赔偿金。拒发实发工资与最低工资标准差额及赔偿金的，对企业处以实发工资与最低工资标准差额及赔偿金１至３倍的罚款。

　　随意加班加点的，应立即改正，不改正的，按超规定总工时数每人当月实得工资的时、日平均数的５倍处以罚款。

　　**第三十条**　企业不为职工办理社会保险手续的，应按照劳动行政部门规定的期限补办；不按期缴纳各项社会保险费的，应当从逾期之日起按日加收应缴金额２‰的滞纳金。滞纳金分别纳入各项社会保险费用。

　　**第三十一条**　企业违反劳动安全卫生规定的，应令其限期改正或停业整顿，并按有关规定处以罚款。

　　**第三十二条**　阻挠或拒绝劳动行政部门进行劳动监察的，处以月经营及销售收入１‰以下的罚款。

　　**第三十三条**　以上各项罚款，当地劳动行政部门应在对其警告后仍不改正的情况下，方可实施。

　　**第三十四条**　上述行政处罚，由劳动行政部门依法执行。罚款全部上交国库。

　　**第三十五条**　华侨和台湾、香港、澳门投资者在中国大陆投资举办的合资经营企业、合作经营企业和拥有全部资本的企业及股份有限公司，均适用本规定。

　　**第三十六条**　本规定由劳动部负责解释。

　　本规定自发布之日起施行。过去有关外商投资企业劳动管理规定与本规定有抵触的，按本规定执行。

© 北大法宝：（[www.pkulaw.com](https://www.pkulaw.com/)) 专业提供法律信息、法学知识和法律软件领域各类解决方案。北大法宝为您提供丰富的参考资料，正式引用法规条文时请与标准文本核对。
欢迎查看所有[产品和服务](https://www.pkulaw.com/helps/2.html)。法宝快讯：[如何快速找到您需要的检索结果？](https://www.pkulaw.com/helps/70.html)[法宝](https://www.pkulaw.com/helps/69.html)V6有何新特色？

扫描二维码阅读原文

**原文链接：**<https://www.pkulaw.com/chl/1782c2a54edd642cbdfb.html>
